# Differential Responses of the Egg-Larval Parasitoid *Chelonus Bifoveolatus* To Fall Armyworm-Induced and Constitutive Volatiles of Diverse Maize Genotypes

**DOI:** 10.1007/s10886-025-01585-3

**Published:** 2025-03-12

**Authors:** Collins O. Onjura, Emmanuel Peter, George O. Asudi, Michael M. Gicheru, Samira A. Mohamed, Toby J. A. Bruce, Amanuel Tamiru

**Affiliations:** 1 International Centre of Insect Physiology and Ecology, P. O. Box 30772-00100, Nairobi, Kenya; 2 Kenyatta University, P. O. BOX 43844-00100, Nairobi, Kenya; 3 Federal University Gashua, P.M.B 1005, Gashua, Yobe State Nigeria; 4 School of Life Sciences, Keele University, Staffordshire, Keele, ST5 5BG UK

**Keywords:** *Spodoptera frugiperda*, *Chelonus bifoveolatus*, Behavioural response, Plant volatiles, Maize genotypes, Pest management

## Abstract

The fall armyworm (FAW), *Spodoptera frugiperda*, is a serious invasive crop pest and threat to food security. Conventional pest control approaches using chemical pesticides can lead to adverse environmental and human health problems calling for safer alternative pest management options. Volatile organic compounds (VOCs) released by plants constitutively and in response to herbivory have been shown to enhance ecologically benign biocontrol alternatives to chemical insecticides for pest management. However, genotypic variations in VOC emissions have also been reported for plant species including maize (*Zea mays*). Hence, a better insight into the variations in odor profiles of different maize varieties and their corresponding role in recruiting pests’ natural enemies are crucial for developing a sustainable biocontrol strategy. Our present study assessed the behavioral responses of the FAW egg-larval parasitoid, *Chelonus bifoveolatus* (Braconidae: Hymenoptera), to constitutive and induced volatiles from different maize landraces (Jowi Red, Nyamula) and hybrids (SC Duma, DK 777) grown in Kenya and compared their volatile profiles. In a four-arm olfactometer, female parasitoid wasps were significantly attracted to FAW oviposition-induced VOCs from SC Duma and Nyamula. Chemical analysis of test plant volatiles revealed significant variation in the quantity and quality of key bioactive VOCs such as (*E*)-2-hexenal, α-pinene, (*Z*)-3-hexenyl acetate, (*E*)-4,8-dimethyl-1,3,7-nonatriene, α-copaene, (*E)*-β-farnesene and (*E*,* E*)-4,8,12-trimethyl-1,3,7,11-tridecatetraene. Our findings provide more insights into genetic variation in VOCs emission across maize genotypes and the corresponding differences in attraction of pest natural enemies that provide indirect defense. As such, these traits could be exploited to enhance ecologically sustainable pest management strategies.

## Introduction

The fall armyworm (FAW). *Spodoptera frugiperda* (Lepidoptera: Noctuidae), is a serious invasive crop pest that poses a significant threat to food security, impacting agricultural production and livelihoods worldwide (Kansiime et al. 2023). It belongs to the genus *Spodoptera*, a section of Noctuidae responsible for the highest monetary losses to agricultural crop production worldwide (Pogue 2002). FAW prefers to feed on cereals, particularly maize (*Zea mays* L.) (Altaf et al. 2022; Sisay et al. 2023), a staple food and cash crop in the African region (Ekpa et al. 2019), with an additional 353 plant species reported as its host including rice, sorghum, wheat, sugarcane, cotton, and vegetable crops (Montezano et al. 2018). The pest is native to the Americas (Jing et al. 2021) but has recently spread to Africa, colonizing and negatively impacting a continent already embattled with food insecurity (Khan et al. 2014; Goergen et al. 2016). In sub-Saharan Africa (SSA), crops estimated to be worth over US $13 billion yearly are at risk of FAW damage (Kansiime et al. 2023), thus putting in danger the lives and livelihoods of millions of smallholder farmers (Harrison et al. 2019). Yield losses ranging from 11 to 58% have been reported for maize alone in different African cropping systems (Chimweta et al. 2020), translating to a loss in revenue of up to US $9.4 billion yearly (Eschen et al. 2021).

In North and South America where the pest is endemic, chemical insecticides and transgenic maize varieties are commonly used to control FAW (Fatoretto et al. 2017). The large-scale commercial farmers in this region offset the high cost of these technologies as they have access to high and stable international markets and are often supported by government subsidies and risk-transfer mechanisms (Hruska 2019). In contrast, most African smallholder farmers are resource-constrained and therefore cannot afford the expensive technologies and repetitive spraying to achieve pesticide efficacy. Moreover, use of transgenic Bt maize is constrained by complicated regulatory requirements and negative campaigning by non-governmental organizations who oppose this technology (Herring 2008). Furthermore, FAW has evolved resistance to Bt after use of transgenic Bt crops in the Americas for an extended period (Banerjee et al. 2017). In addition, control of FAW using pesticides not only causes undesirable risks to human and environmental health (Harrison et al. 2019; Kansiime et al. 2019) but also leads to resistant development and negatively interferes with the ecosystem by killing beneficial insects like natural enemies (Mihm 1997; Kumela et al. 2019). Therefore, devising an environmentally friendly, affordable and sustainable FAW control approach is of prime importance to smallholder farmers in SSA.

Indirect plant defence is an ecologically sustainable strategy that could potentially provide a culturally appropriate and low-cost pest control option (Harrison et al. 2019). This plant defense mechanism is mediated by the production of blends of volatile organic compounds (VOCs) in response to herbivory to attract natural enemies of the herbivore such as parasitoids (War et al. 2012). Parasitoids are well-known biological control agents against arthropod pests (Wang et al. 2019). They depend on olfaction to locate their hosts in complex chemical environments (Sun et al. 2019). In this process of host location, parasitoid wasps rely on the interplay of short- and long-range chemical signals from plants, herbivores and plant-herbivore interactions (Ortiz-Carreon et al. 2019). The plant derived VOCs could be either induced by herbivores or constitutively produced (Turlings et al. 1990; Steinberg et al. 1993; Paré and Tumlinson 1997; Wenke et al. 2010). However, VOCs induced in response to herbivory and/or oviposition show the likely existence of the host pest insects and, therefore, are exploited by parasitic wasps as more reliable cues (Vet and Dicke 1992; Tamiru et al. 2011, 2012). The herbivore-induced plant volatiles (HIPVs) vary qualitatively and quantitatively based on plant age, plant genotype, herbivore strains, the severity of the attack, abiotic factors or a combination of these (Aartsma et al. 2017). Moreover, HIPV blends are often unique and different to the volatile profiles from healthy plants or those that have been mechanically damaged (Ponzio et al. 2014; Silva et al. 2017). Several studies have documented attractiveness of HIPVs to parasitoids compared to chemical cues derived from undamaged or mechanically damaged plants (Peñaflor et al. 2011; Poelman et al. 2012; Tamiru et al. 2015a, b).

The role of both egg-induced and larval-induced volatiles in maize tri-trophic interactions with lepidopteran herbivores and their parasitoids has already been studied (Tamiru et al. 2011, 2015a; Mutyambai et al. 2015, 2016; Christensen et al. 2013; Ortiz-Carreon et al. 2019). These studies have established that maize plants produce semiochemicals in response to both egg deposition and larval (caterpillar) feeding damage that lure female parasitoids that provide biological control of the herbivore. Furthermore, recent studies by Sobhy et al. (2022) and Peter et al. (2023) have shown that scents released constitutively by companion plants can also attract the FAW parasitoids. However, to the best of our knowledge, no study has been conducted to simultaneously investigate the role of oviposition and larval-induced volatiles on egg-larval *C. bifoveolatus* parasitoids. This could also be due to the different strategies employed by egg and larval parasitoids in locating hosts (Roque-Romero et al. 2020). Previous studies on related *Chelonus* species such as *Chelonus insularis* (Hymenoptera: Braconidae), have focused on the parasitoid’s responses to either larval-induced or egg-induced responses but not considered both responses simultaneously (Ortiz-Carreon et al. 2019; Roque-Romero et al. 2020). In addition, strong evidence of variation between maize genotypes in VOC emissions leading to differences in attraction of pest natural enemies (parasitoids) have been recorded (Degen et al. 2004; Tamiru et al. 2015b, 2020; Raglin et al. 2022; Wang et al. 2023). Hence, a more nuanced understanding of the variation in odor profiles of the different maize varieties/genotypes and their ecological benefit in attracting pest’s natural enemies will greatly contribute to developing an effective biological control strategy.

*Chelonus bifoveolatus* (Hymenoptera: Braconidae) is a FAW egg-larval parasitoid belonging to the genus *Chelonus* (Shen et al. 2023) and is a widespread FAW egg-larval parasitoid in Africa (Otim et al. 2021). The parasitoid deposits its eggs inside FAW eggs which, unlike the parasitism by other egg parasitoids, hatch into neonates. However, the host larval development is delayed until it reaches the third instar where the final stage of the parasitoid comes out of the dying host and devours the residues except for the host head capsule (Huddleston and Walker 1994). Similar to other egg-larval parasitoid wasps, *C. bifoveolatus* has the possibility of experiencing a divergent range of semiochemicals in the ecosystem as well as facing the challenge of the low perceptibility of its host eggs. Therefore, the host location abilities of *C. bifoveolatus*, in response to both FAW egg and larval-induced volatiles produced by different maize varieties, would be crucial for its fitness and it is likely to have evolved sophisticated sensory abilities to enable host detection. Here, we observed and assessed responses of *C. bifoveolatus* to both constitutive and FAW-induced maize volatiles from different maize varieties using a four-arm olfactometer. Moreover, we subsequently identified and quantified the headspace VOCs constitutively released by maize plants as well as those released in response to FAW oviposition and larval damage, using GC-MS.

## Methods and Materials

*Experimental Plants.* The commercial hybrid varieties SC Duma 43 and DK777 and maize landraces Jowi Red and Nyamula were selected for the experiment. The commercial hybrids were purchased from Kenya Seed Company Limited, Nairobi, Kenya while maize landraces were sourced from small-scale farmers in Homabay County, Western Kenya. The different maize varieties were grown individually in 4 L plastic pots filled with organic manure and soil in a 1:2 ratio, respectively. The plants were maintained in an insect proof screenhouse under natural conditions, *c.* 25 ± 2 °C, 65 ± 5% RH; 12 L: 12D at the International Centre of Insect Physiology and Ecology (*icipe)*, Duduville, Nairobi, Kenya, (01º13′25.6″S 036º53′49.1″E, 1616 masl) and were used for experiments 30 days after sowing, *c.* 45 cm in height.

*Insects.* The initial population of FAW larvae used for our study was sourced from maize farms infested with FAW in the Mbeere region of Embu County, Kenya (00º42′25.1″S 037º29′0.14″E, 1091 masl). The insects were maintained on a natural diet at *icipe’s* Animal Rearing and Quarantine Unit (25 ± 2 °C, 50–70% RH, 12 L:12D photoperiod). The larvae were reared in ventilated sleeved Perspex cages measuring 60 × 60 × 60 cm. A paper towel was placed at the bottom of each cage to provide an environment for pupation as well as for absorption of excess moisture. Young maize leaves were supplied to the FAW larvae in the cages and replaced with fresh leaves every two days as a diet. The pupae were harvested, placed in Petri dishes lined with moistened pieces of cotton wool, and then put inside a clean ventilated-sleeved Perspex cage (30 × 30 × 30 cm) for moths to eclose. Adult moths were fed using a 10% honey-water solution soaked in cotton wool. Butter papers were introduced into cages containing female moths to serve as oviposition substrates. Eggs were harvested daily from the oviposition substrates, placed in a 30 ml glass vial and sealed with cotton wool till the emergence of neonates. Emerged neonates were then transferred into rearing Perspex cages (60 × 60 × 60 cm) and the process repeated as described above. To ensure colony vigor, the laboratory-reared colony was infused every three months with field-collected insects.

For egg-larval parasitoid, *C. bifoveolatu*s, the beginning colony was obtained from field collected FAW larvae from the Mbeere region of Embu County, Kenya. The colony was maintained on FAW eggs at *icipe*’s insect mass rearing unit at room temperature (25 ± 2 °C), relative humidity of 65 ± 5% and 12HL:12HD photoperiod. To maintain the original insect behavioral characteristics and avoidance of genetic decay, the parasitoid colony was quarterly infused with field-collected insects. Third instar larvae and three days old naïve (not previously exposed to the odor source) gravid FAW moths and parasitoids from the two colonies were used in our experiments.

*Volatile Collection.* Volatiles were collected from FAW infested (larval and egg-induced) and non-infested maize plants for 24 h using the headspace sampling technique as described by Tamiru et al. (2011). Before entrainment, seedlings of the different maize varieties were inoculated with six 3rd instar FAW larvae each and kept inside cages measuring 40 × 40 × 60 cm for 24 h, for induced damage. While, for egg induction, the test plants were kept inside similar cages but exposed to eight gravid female FAW moths to oviposit overnight. A cotton wool moistened with a honey-water solution (10%) and placed inside a Petri dish was introduced into each cage containing insect moths as their diet. The cages were covered with black cotton cloths to facilitate oviposition. Upon oviposition, the exposed test plants, with at least 10 egg batches, were selected for volatile collection. Likewise, the non-infested maize varieties were kept inside similar cages and conditions, but without FAW moths, prior to volatile collection. For each treatment, four plant were used for the volatile collection.

Volatile organic compounds were entrained by channeling clean charcoal-filtered air (flow rate of 500 ml/min), through the inlet port of test plants tenderly covered with oven bags (polyethylene terephthalate), volume 3.2 L, ⁓ 12.5 mm thickness, sterilized at 100 ℃ for 1 h before use and closed at the neck of the plant using adjustable plastic tag pin. The compounds emitted were trapped within PorapakQ filters (0.05 g, 60/80 mesh; Supelco, Bellefonte, USA) put at the outlet port with an airflow of 300 ml/min. After entrainment, the collected volatiles were eluted using 0.5 mL dichloromethane (DCM) into sample vials measuring 2 mL (Agilent Technologies, Warsaw, Poland) and later divided into two aliquots, one for olfactometer bioassay and the other for chemical analysis, and frozen at -40 ℃ before use.

*Four-arm Olfactometer Bioassay.* The responses of FAW egg-larval parasitoids (*C. bifoveolatus*) to plant VOC samples and solvent control were assessed using a Perspex four-arm olfactometer as described by Tamiru et al. (2012) under controlled laboratory conditions of 25 ± 2 °C and 70 ± 5% RH from 10:00 a.m. to 4:00 p.m. Two series of choice test bioassays were conducted to compare the behavioural response of *C. bifoveolatus* to: (i) volatiles from oviposition-induced, constitutive test plants and solvent control (DCM), and (ii) volatiles from larval-induced, constitutive test plants and solvent control. The treatments, i.e., herbivore-induced (egg or larval-infested) volatiles and their respective constitutive volatiles were placed in the two opposite arms of the olfactometer while the remaining two arms had solvent control (DCM). A suction pump, connected to the center of the olfactometer, was used to draw (260 mL/min) air containing the scent from the four arms towards the center of the olfactometer arena. Micropipettes (Drummond microcap, Drummond Scientific Co., Broomall, PA, USA) were used to apply 10 µL aliquots of test VOC samples to a cut piece (4.5 mm x 2.5 mm) of filter paper; Whatman, Maidstone, United Kingdom and then placed in the olfactometer arms. Mated naïve *C. bifoveolatus* females were then individually introduced into the central chamber, using a custom-made piece of plastic tubing, and their movement was evaluated for 12 min. The number of entries and time spent in each arm by individual parasitoids were recorded and summarized using ‘Olfa’ software (F. Nazzi, Udine, Italy). Each female parasitoid was observed in the olfactometer bioassay only once with 12 replications per treatment combination. The position of the treatment arms in the olfactometer alternated clockwise (Tamiru et al. 2011; Peter et al. 2023) every 3 min to avoid orientation bias. In every replicate, a scrupulously clean olfactometer was used and upon completion, all bioassay equipment was washed and cleaned using 70% ethanol and air dried for the next use.

*Chemical Analysis of Volatiles*. Test plant headspace volatile samples (2 µL) were analysed on an Agilent 7890 A gas chromatograph (GC) coupled to a mass spectrometer (MS) (MSD 5975 C triple-axis, Agilent Technologies, Palo Alto, USA) in splitless mode. The GC-MS machine had a non-polar capillary column (HP5-MSI, 30 m length × 0.25 mm i.d. × 0.25 μm film thickness) (J & W Scientific, Folsom, USA), with helium as carrier gas at a 1.2 mL min^− 1^ flow rate. The oven temperature was maintained at 35 °C for 5 min and thereafter programmed to increase at a rate of 10 °C per min to a final temperature of 280 °C and maintained for a duration of 10.5 min. The ion source temperature for the mass selective detector was maintained at 230 ^0^C and the recording of spectra was set at an electron impact factor of 70 eV while the temperature of the MS quadrupole was held at 150 °C (Peter et al. 2023). Volatile compounds were characterized by comparing their mass spectra data with authentic standards in reference databases (Chemecol, NIST11 and Adams2) as well as making use of retention indices of a mixture of n-alkanes (C8–C23) to calculate their retention indices. To validate the tentative GC-MS identifications of volatile compounds, we did co-injections with commercially available authentic standards. Quantification of the identified volatiles (in nanograms) was achieved using external calibration curves derived from 1000 ng µL − ^1^ stock solutions prepared from (*E*)-β-caryophyllene and β-pinene compounds, having varying concentrations ranging from 0.1 to 1000 ng/µl. The concentrations of the VOCs were calculated by dividing the peak areas of the compounds by known amounts of authentic standards and then converted into nanograms emitted per plant per hour (ng/plant/hr). Any peaks that appeared in the blank (polyethylene terephthalate bags) were considered as contaminants and thus discarded from the quantification analysis. The data generated were analysed with the MSD Chemstation software F.01.00.1903 (Agilent Technologies).

*Chemicals.* Dichloromethane (99.9% purity) used in our experiments was purchased from Merck (Darmstadt, Germany). (Z)-3-hexen-1-ol, α-pinene, (*E*)-2-hexenal, 2-heptanol, β-pinene, β-myrcene, α-humulene, limonene, (*E*)-β-ocimene, linalool, and (*E*)-β-caryophyllene (authentic standards with a purity of > 95%) were sourced from Sigma-Aldrich and used for confirmation of tentative GC-MS identifications as well as in quantification analysis.

*Statistical Analyses.* All generated data were analyzed using R statistical software version 4.0.4 (R Core Team 2021). Data from *C. bifoveolatus* olfactometer responses (time spent) were not normally distributed (Shapiro-Wilk test: *p* < 0.05); therefore, we used Kruskal-Wallis’s test (non-parametric) for the analysis upon conversion of the data into proportions, to account for dependence of visiting time by *C. bifoveolatus* within the fields of the olfactometer, then log_10_-ratio transformed to enable analysis of compositional data in accordance to Tamiru et al. (2011) and Aitchison (1982). We then used Dunn’s post-hoc test to separate significant means. The concentrations of VOCs produced by the treatments (test plants) were analysed using Mann-Whitney Wilcoxon and Kruskal-Wallis tests for two treatments and multiple treatments respectively because the data were not normally distributed (Shapiro-Wilk test: *p* < 0.05) and significant means separated using Dunn’s post-hoc test. To find the relative contribution of differentially emitted VOCs to dissimilarity across the experimental plants, we did a similarity percentage analysis (SIMPER) by subjecting the volatile organic compounds peak areas to SIMPER analysis. We then used non-metric multidimensional scaling (NMDS) as well as one-way ANOSIM with the Bray-Curtis dissimilarity matrix, respectively, to envision the profile and compare the volatile organic compounds profiles of the twelve experimental plants.

## Results

**Fig. 1:**
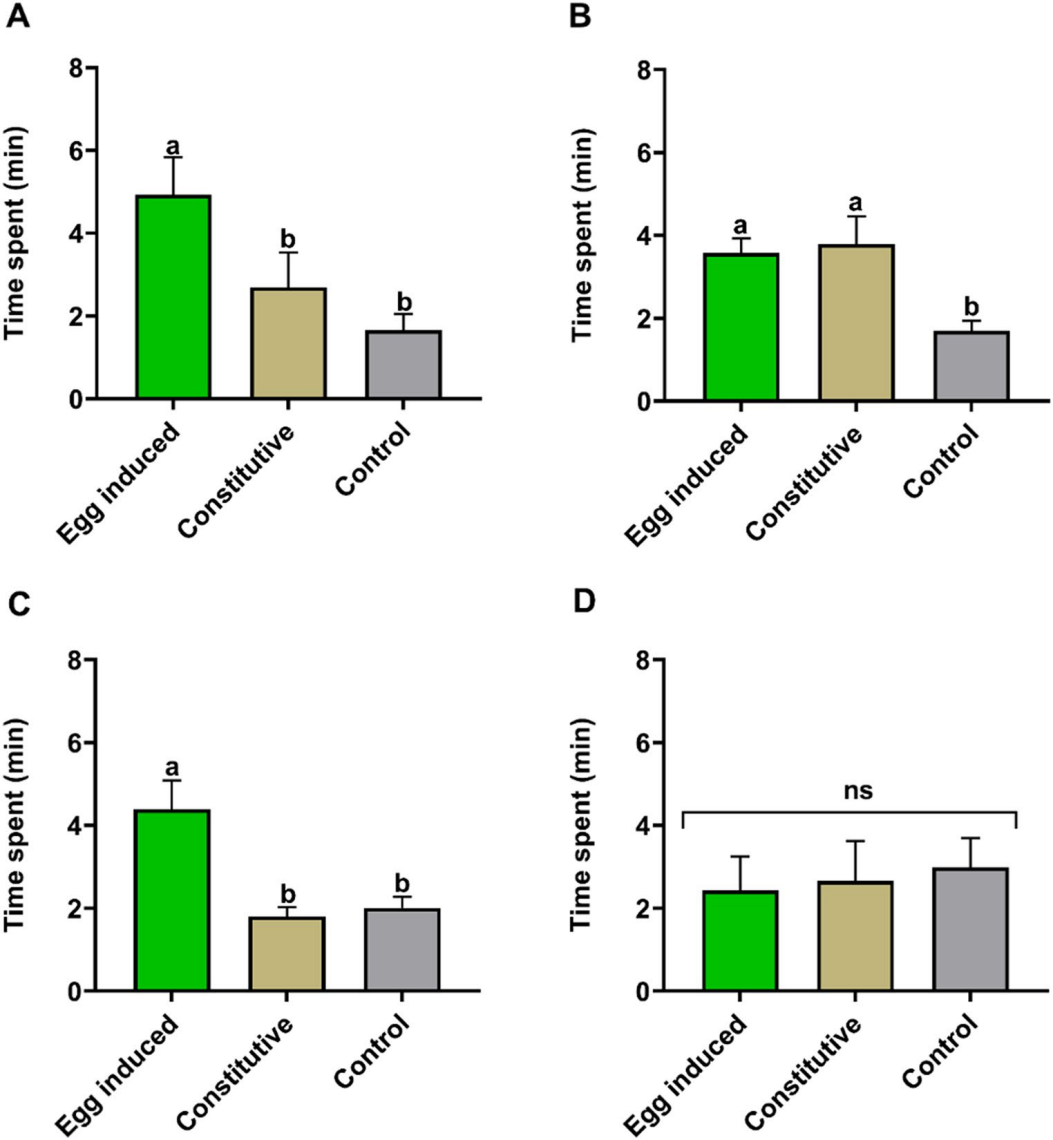
Behavioral response of female *Chelonus bifoveolatus* to *Spodoptera frugiperda* egg-induced and constitutive headspace volatiles from four maize, *Zea mays*, varieties (**A**) SC Duma, (**B**) Jowi Red, (**C**) Nyamula, (**D**) DK 777 in a four-arm olfactometer bioassay. Individual female parasitoids were monitored for 12 min (*N* = 12). Bars indicate time spent (minutes; mean ± SE) by *C. bifoveolatus* in different regions of the olfactometer. Control = solvent (DCM) only; ns = no significant difference in time spent

*Response of C. bifoveolatus to Oviposition-Induced and Constitutive Maize Volatiles.* Behavioral responses of *C. bifoveolatus* females to egg-induced and constitutive maize VOCs, and solvent control (DCM) are shown in Fig.  1. The parasitoid wasps were significantly attracted to oviposition-induced plant volatiles from SC Duma (Kruskal–Wallis χ^2^ = 14.162, df = 2, *P* < 0.001; Fig.  1A) and Nyamula (Kruskal–Wallis χ^2^ = 14.696, df = 2, *P* < 0.001; Fig.  1C) compared to their respective constitutive volatiles and solvent control (Fig.  1A, C). Interestingly, the parasitoids were equally attracted to constitutive and oviposition-induced volatiles from Jowi Red landrace (Fig.  1B) though time spent in the olfactometer areas with these volatiles was higher than time spent in the solvent control area (Kruskal–Wallis χ^2^ = 19.613, df = 2, *P* < 0.001; Fig.  1B). However, *C. bifoveolatus* females were not able to discriminate between the different odour sources when DK 777 was tested (Kruskal–Wallis χ^2^ = 0.19367, df = 2, *P* = 0.9077; Fig.  1D).

**Fig. 2:**
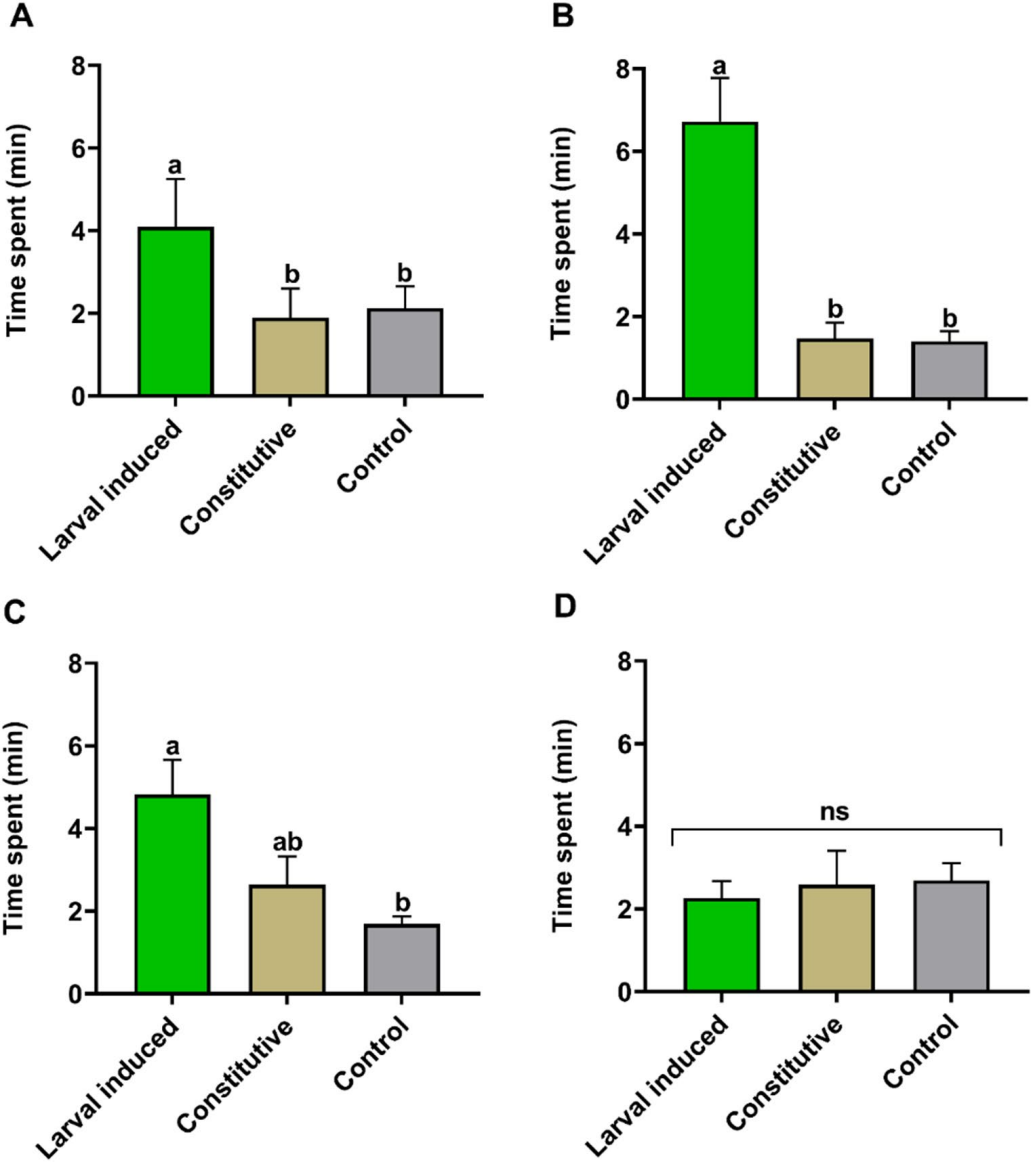
Behavioral response of female *Chelonus bifoveolatus* to *Spodoptera frugiperda* larval-induced and constitutive headspace volatiles of four maize, *Zea mays*, varieties (**A**) SC Duma, (**B**) Jowi Red, (**C**) Nyamula, (**D**) DK 777 in a four-arm olfactometer bioassay. Individual parasitoids were monitored for 12 min (*N* = 12). Bars indicate time spent (minutes; mean ± SE) by *C. bifoveolatus* in different regions of the olfactometer. Control = solvent (DCM) only; ns = no significant difference in time spent

*Response of C. bifoveolatus to Larval-Induced and Constitutive Maize Volatiles.* In this experiment, the parasitoid wasps were significantly attracted to larval-induced plant volatiles from SC Duma (Kruskal–Wallis χ^2^ = 7.4048, df = 2, *P* = 0.02; Fig.  2A) and Jowi Red (Kruskal–Wallis χ^2^ = 21.153, df = 2, *P* < 0.001; Fig.  2B), compared to their respective constitutive volatiles and control (DCM) (Fig.  2A, B). We also observed that the parasitoids were significantly attracted to larval-induced plant volatiles from Nyamula (Kruskal–Wallis χ^2^ = 12.045, df = 2, *P* = 0.002; Fig.  2C) compared to solvent control. However, no significant difference in the parasitoid response was observed between larval-induced and constitutive DK 777 maize volatiles (Kruskal–Wallis χ^2^ = 1.1454, df = 2, *P* = 0.564; Fig.  2D).

**Fig. 3:**
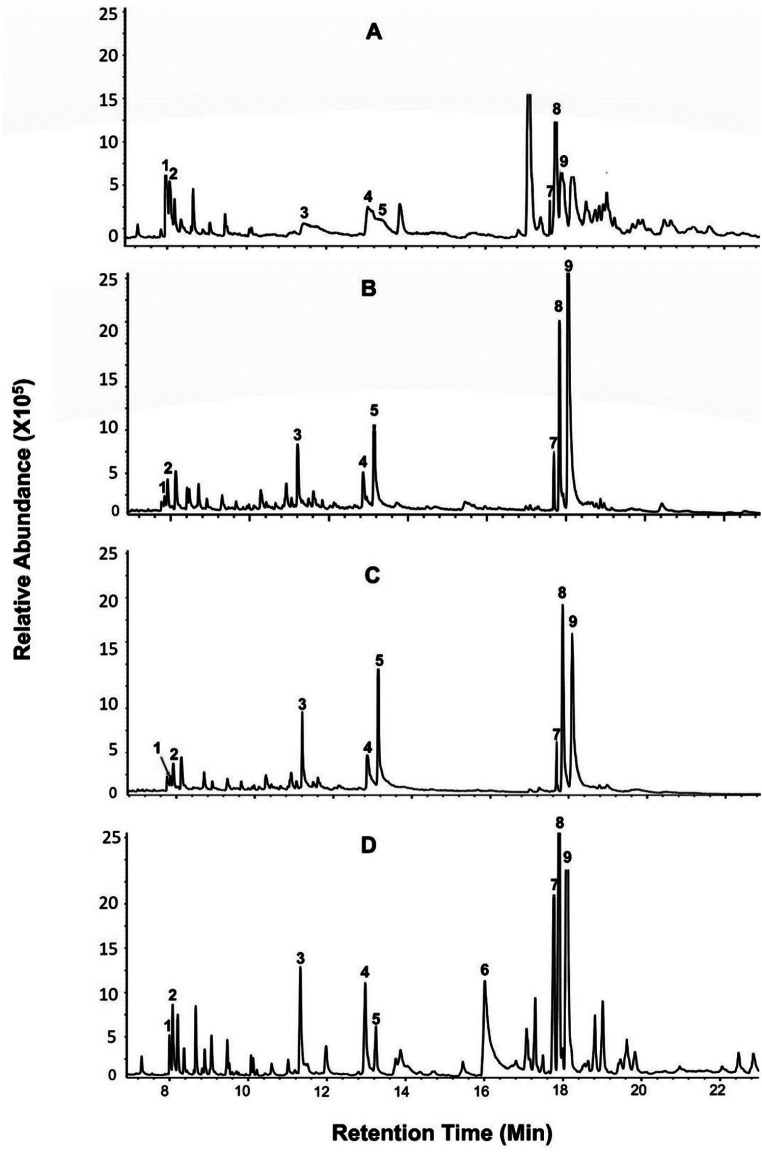
GC-MS profiles of representative headspace samples from *Spodoptera frugiperda* larval-induced maize, *Zea mays*, varieties: (**A**) DK777 (**B**) Nyamula, (**C**) Jowi Red, and (**D**) SC Duma. The labeled GC peaks indicate variations between the maize genotypes in the emission profiles of selected herbivore-induced plant volatiles reported to confer defense against herbivore pests. (1) (*E*)-2-hexenal, (2) (*Z*)-3-hexenol, (3) (*Z*)-3-hexenyl acetate, (4) linalool, (5) DMNT, (6) indole, (7) (*E*)-β-caryophyllene, (8) (*E*)-*α*-bergamotene and (9) (*E*)-β*-*farnesene

*Analyses of Volatile Organic Compounds.* GC-MS analysis of headspace volatiles showed both qualitative and quantitative variation in the volatile profiles of the plants (Table 1; Fig.  3). The analysis identified 33 volatiles belonging to different chemical classes, namely aldehydes [(*Z*)-3-hexenal, (*E*)-2-hexenal, decanal]; alcohols [(*Z*)-3-hexenol, (*E*)-2-hexenol, 2-heptanol]; benzenoids (indole); esters [(*Z*)-3-hexenyl acetate]; homoterpenes [(*E*)-4,8-dimethyl-1,3,7-nonatriene (DMNT), (*E*,* E*)-4,8,12-trimethyl-1,3,7,11-tridecatetrae (TMTT)]; monoterpenes [(α-pinene, β-pinene, β-myrcene, limonene, β-ocimene, linalool, camphor); and sesquiterpenes [cyclosativene, α*-*ylangene, β-elemene, α*-*copaene, (*E*)-β-caryophyllene, (*E*)-β*-*farnesene, (*E*)-*α*-bergamotene, *α-*humulene, germacrene D, β-selinene, *α-*selinene, β-bisabolene, *α-*muurolene, γ-cadinene, β-sesquiphellandrene, δ-cadinene]. Among the volatiles detected, the sesquiterpene (*E*)-β*-*farnesene identified from larvae induced Nyamula sample was the most abundant VOC emitted, followed by (*E*)-*α*-bergamotene and (*E*)-β-caryophyllene in larval damaged SC Duma (Table 1).

The four maize genotypes emitted varying amounts of bioactive volatiles, while some VOCs elevated in response to FAW larval feeding and oviposition. For example, after FAW larval feeding, the abundance of (*E*)-β*-*farnesene was significantly higher in Nyamula headspace volatiles compared to Jowi red, SC Duma and DK 777 plants and the undamaged Nyamula control plants themselves (Kruskal–Wallis χ2 = 20.973, df = 7, *P* = 0.003; Table 1). (*E*)-*α*-bergamotene was strongly induced in larval damaged SC Duma samples compared to Nyamula, DK 777 and healthy SC Duma control volatiles (Kruskal–Wallis χ2 = 32.706, df = 11, *P* < 0.001; Table 1). Similarly, significantly higher levels of (*E*)-β-caryophyllene were recorded in volatiles from larval damaged SC Duma compared to Jowi red, Nyamula, DK 777 and undamaged SC Duma control (Table 1). In responses to FAW oviposition, DMNT and (*E*)-2-hexenal were strongly induced by SC Duma compared to uninfested control. Similarly, DMNT and the monoterpene β-myrcene detected from Nyamula egg-induced samples were the most abundant among egg-induced volatiles in Nyamula (Kruskal–Wallis χ2 = 16.794, df = 8, *P* = 0.03; Table 1).

The homoterpene TMTT (Kruskal–Wallis χ2 = 20.973, df = 7, *P* = 0.003) was identified in both larval and egg-induced samples from all the four maize varieties used in this study, however, it was not detected in any of the control samples and was found in significantly lower amounts in DK777. The sesquiterpene *α-*copaene (Kruskal–Wallis χ2 = 24.667, df = 8, *P* = 0.001), with varying quantities, was detected in samples from all the treatments of SC Duma, Jowi Red and Nyamula; however, *α-*copaene was not found in any of the samples from DK777 variety hence could be of potential biological relevance. Similarly, another sesquiterpene, *α-*ylangene (Kruskal–Wallis χ2 = 4.3556, df = 2, *P* = 0.11) was identified in all treatments of DK 777 maize variety but not in the treatments of the other three varieties. (*E*)-β-caryophyllene, *α*-pinene, limonene, linalool, cyclosativene, DMNT, and (*E*)-*α*-bergamotene volatiles were identified in treatments from all maize varieties studied with significant differences across the treatments. The alcohol (*E*)-2-hexenol was only identified in SC Duma and Jowi Red larval-induced samples. The monoterpenes β-ocimene and camphor were found in both SC Duma larval and egg-induced samples and in Jowi control and Nyamula egg-induced samples, respectively. The benzenoid indole was only detected in the SC Duma larval-induced samples (Table 1).

**Table 1:** Mean amounts of identified volatiles in ng/plant/h of the headspace extracts from *Spodoptera frugiperda* induced and health maize, *Zea mays*, varieties

Peak no.	Compound	SCL	SCE	SCC	JowiL	JowiE	JowiC	NyamL	NyamE	NyamC	DK777L	DK777E	DK777C	*P* value
1	(*Z*)-3-hexanal	29.56 ± 3.9bF	58.98 ± 5.52aA	nd	10.94 ± 0.52dF	11.32 ± 1.76dC	12.73 ± 1.24dE	11.18 ± 0.52dE	21.47 ± 4.51cC	8 ± 0.57dC	11.24 ± 1.62dC	nd	7.28 ± 0.3dB	0.004
2	*(E*)-2-hexenal	61.44 ± 8.22aE	50.2 ± 9.3aA	nd	12.27 ± 0.49bF	nd	nd	11.08 ± 0.3bE	10.77 ± 0.32bD	nd	18.08 ± 7.97bC	nd	nd	0.02
3	(Z)-3-hexenol	42.66 ± 2.53bE	50.71 ± 8.17bA	8.47 ± 0.74cB	12.72 ± 1.95cF	nd	11.06 ± 0.93cE	11.2 ± 0.5cE	13.75 ± 0.06cD	9.42 ± 1.01cC	67.95 ± 8.18aA	11.51 ± 2.41cC	8.94 ± 0.13cB	**0.004**
4	*(E*)-2-hexenol	12.69 ± 4.11aF	nd	nd	14.38 ± 1.32aF	nd	nd	nd	nd	nd	nd	nd	nd	0.7
5	2-Heptanol	nd	7.37 ± 0.37bD	8.08 ± 0.68bB	12.31 ± 0.58bF	nd	nd	11.6 ± 1.99bE	nd	21.42 ± 2aA	nd	nd	nd	0.02
6	α-Pinene	10.31 ± 0.49cF	17.54 ± 0.63bD	8.32 ± 1.42cB	12.48 ± 0.2cF	8.08 ± 0.1cC	34.18 ± 0.71aB	11.51 ± 0.43cE	20.61 ± 1.93bC	10.8 ± 0.76cB	18.23 ± 4.62bC	12.2 ± 3.08cC	10.82 ± 1.45cB	0.01
7	β-Pinene	10.25 ± 0.09bF	7.07 ± 0.27bD	nd	11.71 ± 0.24bF	8.68 ± 0.32bC	18.66 ± 1.51aD	11.5 ± 0.33bE	12.29 ± 0.71bD	10.63 ± 0.38bB	nd	14.28 ± 4.52aC	nd	0.01
8	β-Myrcene	14.18 ± 0.24cF	14.02 ± 6.76cD	7.19 ± 0.22dB	19.03 ± 0.37cF	nd	26.06 ± 2.63cC	16.31 ± 5.17cE	72.93 ± 5.24aA	nd	15.24 ± 4.47cC	34.38 ± 10.19bB	nd	0.03
9	(*Z)-3-*hexenyl acetate)	78.03 ± 7.32aD	33.16 ± 2.07cC	7.71 ± 0.81eB	50.88 ± 1.35bD	nd	24.43 ± 0.9dC	46.73 ± 5.71bD	14.55 ± 2.04eD	8.38 ± 0.87eC	20.53 ± 6.92dC	nd	nd	**0.002**
10	Limonene	10.21 ± 1.2aF	19.75 ± 9.61aD	12.49 ± 3.61aB	15.35 ± 3.18aF	14.94 ± 1.38aC	30.48 ± 3.65aB	13.77 ± 3.57aE	24.81 ± 2.2aC	12.48 ± 2.24aB	13.28 ± 1.26aC	13.16 ± 4.17aC	19.3 ± 2.57aA	0.11
11	β-ocimene	9.61 ± 1.92aF	10.37 ± 3.72aD	nd	nd	nd	nd	nd	nd	nd	nd	nd	nd	0.7
12	Linalool	48.34 ± 1.04aE	11.96 ± 4.83cD	11.72 ± 3.14cB	35.34 ± 3.2bE	25.05 ± 1.85bC	27.93 ± 6.56bC	22.06 ± 6.29bE	37.35 ± 1.3bB	24.99 ± 5.48bA	20.48 ± 6.34bC	19.68 ± 2.49bC	19.69 ± 3.22bA	0.01
13	DMNT	56.55 ± 0.75cE	54.97 ± 2.07cA	12.91 ± 4.3dB	120.95 ± 4.44aB	25.34 ± 2.43dC	51.5 ± 7.54cA	89.24 ± 7.92bB	64.01 ± 3.03cA	11.94 ± 1.39dB	24.08 ± 7.02dC	19.04 ± 7.99dC	26.73 ± 6.02dA	**0.001**
14	camphor	nd	nd	nd	nd	nd	11.44 ± 1.82aE	nd	13.46 ± 3.4aD	nd	nd	nd	nd	1
15	Decanal	13.47 ± 1.98aF	nd	nd	7.88 ± 0.25aF	18.09 ± 7.65aC	nd	9.03 ± 1aE	17.15 ± 7.7aD	13.13 ± 3.66aB	nd	nd	nd	0.52
16	Indole	81.04 ± 10.09D	nd	nd	nd	nd	nd	nd	nd	nd	nd	nd	nd	
17	Cyclosativene	38.54 ± 1.62bE	7.53 ± 0.62dD	19.22 ± 3.66cB	8 ± 0.44dF	22.64 ± 1.6cC	11.19 ± 1.31dE	8.2 ± 1.47dE	13.16 ± 1.17dD	28.58 ± 4.28cA	83.72 ± 8.68aA	21.6 ± 0.58cC	22.7 ± 5.06cA	**0.001**
18	α-Ylangene	nd	nd	nd	nd	nd	nd	nd	nd	nd	26.29 ± 1.14aC	36.13 ± 1.74aB	22.93 ± 6.63aA	0.11
19	α-Copaene	51.28 ± 5.71aE	42.11 ± 2.17aB	19.56 ± 2.68cB	26.84 ± 2.68cE	13.95 ± 0.72dC	20.11 ± 0.48cD	47.12 ± 2.39aD	32.73 ± 2.06bB	8.72 ± 0.31eC	nd	nd	nd	**0.002**
20	β-Elemene	22.72 ± 0.3aF	10.08 ± 2.1aD	10.97 ± 3.21aB	11.56 ± 2.02aF	10.51 ± 1.03aC	19.67 ± 2.63aD	8.84 ± 0.8aE	13.24 ± 5.49aD	11.16 ± 2.16aB	nd	nd	nd	0.20
21	(*E*)- β-Caryophyllene	136 ± 13.57aC	35.4 ± 0.1dB	32.96 ± 2.19dA	81.61 ± 3.83bC	21.22 ± 0.48dC	48.88 ± 3.88cA	42.54 ± 2.27cD	13.35 ± 3eD	13.56 ± 0.39eB	21.22 ± 4.54dC	47.46 ± 10.93cA	10.16 ± 0.15eB	**0.001**
22	(*E)*-α-Bergamotene	173.33 ± 9.4aA	31.67 ± 0.23cC	7.69 ± 1.14dB	156.28 ± 18.84aA	13.18 ± 0.41dC	27.61 ± 3.85cC	63.93 ± 8.49bC	11.28 ± 0.63dD	10.35 ± 1.94dB	50.57 ± 7.45bB	16.41 ± 6.14dC	24.6 ± 3.57cA	**0.001**
23	*(E*)- β-Farnesene	155.45 ± 15.7bB	39.87 ± 0.47cB	nd	155.73 ± 7.5bA	nd	nd	250.3 ± 2.36aA	32.23 ± 2.07cB	25.17 ± 9.34cA	27.13 ± 2.03cC	10.27 ± 0.76cC	nd	**0.004**
24	α-Humulene	18.46 ± 4.68bF	7.02 ± 0.38bD	10.17 ± 1.28bB	nd	14.55 ± 6.15bC	44.64 ± 4.57aA	nd	13.07 ± 3.71bD	16.65 ± 5.41bB	nd	18.43 ± 7.37bC	26.24 ± 8.04bA	0.04
25	β-Selinene	nd	nd	nd	nd	nd	nd	nd	15.99 ± 4.51aD	nd	25.64 ± 6.28aC	nd	nd	0.4
26	Germacrene D	20.86 ± 2.01bF	10.55 ± 2.91bD	16.89 ± 4.61bB	10.96 ± 1.27bF	36.73 ± 3.24aB	12.35 ± 2.33bE	13.65 ± 1.76bE	11.9 ± 2.51bD	12.16 ± 1.17bB	nd	41.51 ± 0.25aA	24.08 ± 6.14bA	0.02
27	α-Selinene	nd	nd	nd	nd	nd	nd	nd	nd	11.39 ± 1.97B	nd	nd	nd	
28	α-Muurolene	13.6 ± 1.75bF	8.06 ± 0.94bD	9.16 ± 2.2bB	11.22 ± 0.26bF	41.57 ± 3.7aB	nd	11.37 ± 1.12bE	13.12 ± 2.33bD	8.67 ± 0.38bC	nd	12.23 ± 1.5bC	nd	0.04
29	β-Bisabolene	28.07 ± 8.43bF	9.23 ± 1.36bD	nd	17.08 ± 3.43bF	23.14 ± 6.91bC	45.08 ± 8.89aA	9.52 ± 1.84bE	9.82 ± 0.74bD	12.25 ± 1.53bB	26.77 ± 5.53bC	15.55 ± 5.39bC	nd	0.02
30	γ-Cadinene	nd	nd	7.62 ± 0.6bB	nd	25.31 ± 7.67aC	29.39 ± 3.49aB	nd	9.83 ± 1.02bD	7.52 ± 0.57bC	23.75 ± 4.33aC	27.57 ± 6.15aB	25.37 ± 7.09aA	0.02
31	β-Sesquiphellandrene	41.77 ± 3.42aE	10.91 ± 2.41bD	nd	17.3 ± 4.49bF	nd	22.17 ± 3.71bC	12.42 ± 1.23bE	20.49 ± 6.57bC	nd	nd	nd	nd	0.04
32	δ-Cadinene	nd	nd	19.76 ± 5.75aB	nd	68.93 ± 1.73aA	23.14 ± 5.19aC	nd	nd	13.92 ± 1.21aB	nd	28.81 ± 5.35aB	17.3 ± 1.88aA	0.06
33	TMTT	48.97 ± 4.94aE	36.05 ± 0.71cB	nd	43.78 ± 1.29abD	8.68 ± 0.4eC	nd	41.67 ± 0.2bD	17.78 ± 0.46dD	nd	7.91 ± 0.2eC	8.41 ± 0.43eC	nd	0.003
	**P value**	< 0.001	< 0.001	0.02	< 0.001	0.001	< 0.001	< 0.001	0.001	0.01	0.01	0.01	0.02	

^1^Tentative identification of volatiles was performed by comparing their mass spectra with those from authentic standards where available, mass spectra databases (Adams2, Chemeco, NIST 11) and the online NIST Chemistry WebBook and retention index (KI). The compounds are arranged in order of increasing retention time

^*2*^*P* value of non-parametric Kruskal-Wallis and two samples Wilcoxon test for comparing the amount of volatiles from the test plants. Means (± SE) followed by different letter(s) within a column (upper case letter) and within a row (lower case letter) are significantly different at (*P* < 0.05).*nd* not detected, *SCL:* SC Duma larval, *SCE*: SC Duma egg, *SCC*: SC Duma control, *JowiL:* Jowi Red larval, *JowiE:* Jowi Red egg, *JowiC:* Jowi Red control, *NyamL:* Nyamula larval, *NyamE:* Nyamula egg, *NyamC:* Nyamula control, *DK777L:* DK777 larval, *DK777E:* DK777 egg, *DK777C:* DK777 control, *DMNT=* (*E*)-4,8-Dimethyl-1,3,7-nonatriene, *TMTT*= (*E*,* E*)-4,8,12-Trimethyl-1,3,7,11-tridecatetraene

**Fig. 4:**
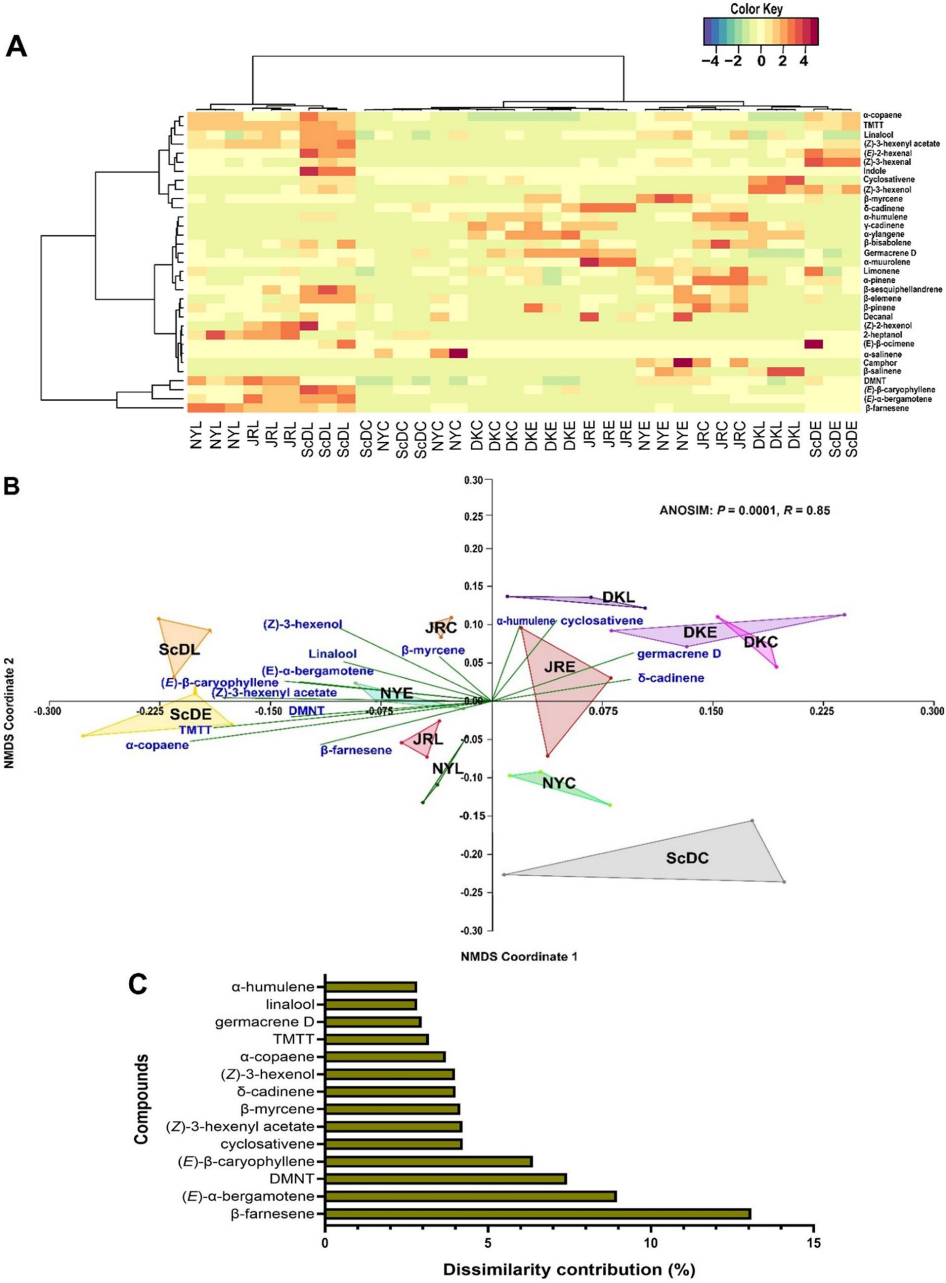
Variations in volatile organic compounds emission between the experimental maize, *Zea mays*, varieties. (**A**) the abundance of volatiles identified as depicted by Heatmap clustering across treatments (**B**) Non-metric multidimensional scaling (distance-Bray; Stress value = 0.21) clustering indicating variations in volatile trends between test plants (**C**) the percentage distribution of the predominant volatile organic compounds from the treatments through analysis of similarities and presented using histogram. *TMTT* = (*E*,* E*)-4,8,12-Trimethyl-1,3,7,11-tridecatetraene, *DMNT* = (*E*)-4,8-Dimethyl-1,3,7-nonatriene. ScDL: SC Duma larval; ScDE: SC Duma egg; ScDC: SC Duma control; JRL: Jowi Red larval; JRE: Jowi Red egg; JRC: Jowi Red control; NYL: Nyamula larval; NYE: Nyamula egg; NYC: Nyamula control; DKL: DK777 larval; DKE: DK777 egg; DKC: DK777 control

Heatmap clustering (Fig. 4A) showed the differences in semiochemical emissions of the experimental plants. The clustering of VOCs emitted across the four maize varieties using a Non-Metric Multidimensional Scaling Plot (NMDS) indicated significant variation between the experimental plants (ANOSIM: *P* = 0.0001, *R* = 0.85) (Fig. 4B). According to the analysis of similarities (ANOSIM), (*E*)-*α-*farnesene (13.1%), (*E*)-*α*-bergamotene (9.0%), DMNT (7.4%), (*E*)-β-caryophyllene (6.4%), cyclosativene (4.2%), (*Z*)-3-hexenyl acetate (4.2%), β-myrcene (4.1%), δ-cadinene (4.0%), (*Z*)-3-hexenol (4.0%), α*-*copaene (3.7%), TMTT (3.2%), germacrene D (3.0%), linalool (2.8%), and *α-*humulene (2.8%) accounted the most for the variations in volatile profiles between the experimental plants (Fig. 4C).

## Discussion

Plant volatile compounds play a crucial role in mediating complex interactions among plants, herbivorous insects and their natural enemies (Bruce et al. 2005; Zhou and Jander 2022). Our results revealed significant attraction of the egg-larval parasitoid, *C. bifoveolatus*, to FAW oviposition and larval-induced maize volatiles. While previous studies showed attraction of other parasitoids to volatiles from herbivore induced maize volatiles, this is the first study to simultaneously consider the effects of larval and egg-induced volatiles from landrace and hybrid maize varieties on *C. bifoveolatus* behavior. Chewing herbivore larvae (lepidopteran caterpillars) not only cause extensive physical damage to the plant but also inject herbivore-associated molecules in their saliva and so it is perhaps not surprising that plant volatile emissions change because of this. In contrast, moth eggs have a more subtle effect on the plant, but we have shown that certain maize genotypes can detect chemicals associated with insect eggs (Tamiru et al. 2011, 2012, 2020).

In this study, we have also observed variation in the *C. bifoveolatus* responses to volatiles from the different maize varieties tested. Female *C. bifoveolatus* were more attracted to FAW egg and larval-induced volatile cues from SC Duma, Nyamula and Jowi Red maize varieties. Interestingly, in the case of DK 777 hybrid maize variety, the parasitoids were not able to discriminate between odor cues from egg and larval-induced plant volatiles (HIPVs), constitutive and control (solvent) treatments. Earlier studies have reported variations in the HIPVs emission by different maize varieties or genotypes leading to differential responses in parasitoids host foraging behavior (Degen et al. 2004; Tamiru et al. 2011, 2015b, 2020; Wang et al. 2023). Several studies have reported intricate and dynamic defense mechanisms evolved by plants which enable them to attract natural enemies of their damaging pests by releasing defense HIPVs (Dicke and van Loon 2000). While it is of adaptive value for the plants to emit HIPVs, parasitoids have also developed the ability to exploit HIPVs as trustworthy chemical cues to identify the presence of their potential hosts and hence enhance their foraging efficiency and ecological fitness.

Our study also revealed qualitative and quantitative variations in the volatile profiles across experimental plants. This agrees with previous studies which showed variations in volatile emissions due to plant species and genotype differences (Gouinguené et al. 2003; Degen et al. 2004; Tamiru et al. 2011). Moreover, chemical analysis of our test plant’s headspace samples revealed elevated levels of volatiles in response to FAW feeding and oviposition. Some of the predominant volatiles identified in our analysis include (*E*)-α-farnesene, (*E*)-*α*-bergamotene, DMNT, (*E*)-β-caryophyllene, (*Z*)-3-hexenyl acetate, β-myrcene, (*Z*)-3-hexenol, *α-*copaene, TMTT, and linalool. Our results are in line with early studies which reported similar compounds, including *α-*pinene, (*E*)-2-hexenal, β-ocimene, (*Z*)-3-hexenyl acetate, linalool, indole, *α-*copaene, (*E*)-β-caryophyllene, DMNT, (*E*)-*α-*farnesene and TMTT as semiochemical cues used by parasitoids for herbivore location and oviposition (Tamiru et al. 2015a, b; Richter et al. 2016; Magara et al. 2020). The increased HIPVs emission following herbivory or oviposition enables natural enemies to distinguish plants colonized by their hosts; hence, serving as an indirect form of defense by plants (Gouinguené et al. 2003; Martorana et al. 2019). Similarly, several previous studies have established that parasitoid wasps are attracted to plant volatiles induced by herbivory or oviposition of their host (Turlings et al. 1990; Steinberg et al. 1993; Wenke et al. 2010; Peñaflor et al. 2011; Tamiru et al. 2011, 2012). The lack of discrimination by female *C. bifoveolatus* to induced and non-induced volatile cues from DK 777 hybrid maize could be due to the fact that this maize variety lacked discernable HIPVs that the parasitoid could exploit for host finding, most likely because it had less induced defense. For example, key HIPVs such as DMNT and TMTT, which have been reported to elicit bioactivity, were induced in significantly lower amounts in DK777 compared to the other maize varieties tested. Incidence of loss of direct and indirect defense traits have been reported in earlier studies (Sotelo 1997; Rasmann et al. 2005; Köllner et al. 2008; Tamiru et al. 2011), corroborating our current findings.

In a recent study, we identified bioactive VOCs from companion crops used in push-pull cropping systems that repel FAW moths and attract its natural enemies *Cotesia icipe* (Hymenoptera: Braconidae) and *Coccygidium luteum* (Hymenoptera: Braconidae) (Sobhy et al. 2022). *Coccygidium luteum* showed electrophysiological responses to TMTT, β-caryophyllene, indole, methyl salicylate (MeSA), (*E*,* E*)-allo-ocimene, DMNT, (*S*)-linalool, β-ocimene, (*Z*)-3-hexenyl acetate, 1-octen-3-ol and (*E*)-2 hexenal, while *C. icipe* to TMTT, β-selinene, MeSA, DMNT, (*S*)-linalool, β-ocimene, (*Z*)-3-hexenyl acetate, 1-octen-3-ol and (*E*)-2-hexenal. In the current study, we identified most of these bioactive VOCs in our treatments. The bioactive homoterpene DMNT was identified in all the treatments; however, TMTT was only detected in larval and egg-induced samples of our treatments. Previous studies have reported strong attraction of braconid parasitoids to these homoterpenes (Khan et al. 1997a; Turlings et al. 1991; Mutyambai et al. 2015; Tamiru et al. 2015a, b). The green leaf volatile (*Z*)-3-hexenyl acetate previously indicated to elicit an electrophysiological response in *Cotesia sesamiae* (Hymenoptera: Braconidae), *B. fusca* and *C. partellus* endoparasitoids (Ngi-Song et al. 2000; Gouinguené et al. 2005; Bruce et al. 2010) was detected in all the treatments except egg-induced samples from Jowi Red and both egg-induced and constitutive volatile samples from DK777 maize. However, (*Z*)-3-hexenyl acetate may not be necessarily an attractant for parasitoid wasps. There can be an electrophysiological response when there is a repellent effect too. Bruce et al. (2010) found (*Z*)-3-hexenyl acetate reduced attraction when there was a larger ratio of it to other HIPVs.

Besides the phenotypic variation, it is plausible that these volatile compounds could play a significant role in mediating *C. bifoveolatus* responses to maize genotypes. The volatiles (*E*)-2-hexenal, linalool, and β-caryophyllene were both detected in our larval and egg-induced samples just as documented in earlier studies (Turlings et al. 1991; Kigathi et al. 2009; Tamiru et al. 2011) and serve in recruiting parasitoid natural enemies (Turlings et al. 1991; De Moraes et al. 1998; Tamiru et al. 2015a). Interestingly, the sesquiterpene *α*-copaene, one of the bioactive compounds in a blend that attracted *C. insularis*, a FAW egg-larval parasitoid (Ortiz-Carreon et al. 2019), was detected in the headspace samples from SC Duma, Jowi Red, and Nyamula. However, it was not identified in any of the DK777 samples, an indication that it could be among the compounds that play a key role in *C. bifoveolatus* attraction to maize derived volatiles. Moreover, it has been established that differences in VOC emissions among maize genotypes, can influence the foraging behaviour of parasitoids in locating their host (Raglin et al. 2022; Tamiru et al. 2020; Wang et al. 2023). In a genome wide association study with 146 maize genotypes, we found that an egg-induced parasitoid attraction trait was more common in landraces than in improved inbred lines and hybrids (Tamiru et al. 2020). Moreover, the study revealed specific genetic variations in the VOC composition and release rates. These changes in VOC mixtures can influence the behavioral response and choices of *C. bifoveolatus* to the different maize plant varieties evaluated as seen in the case of the DK 777 hybrid maize.

Enhancing the attraction of pest natural enemies to herbivore-infested crops can reduce pest damage and improve cropping resilience. Utilization of herbivore-induced VOCs and natural enemies, such as parasitoids as a pest control measure may provide a sustainable as well as an ecologically sound approach to managing devastating pests like FAW. Our study shows that both FAW egg-induced as well as larval-induced VOCs from maize plants attract *C. bifoveolatus*, a FAW egg-larval parasitoid. However, this important defense strategy might have been lost in some of the hybrid maize varieties. Maize is a genetically variable crop, and certain open-pollinated cultivars of Latin American origin were shown to possess VOC mediate defense traits that are not present in the common commercial cultivars (Tamiru et al. 2012). Our current findings provide more insights into the role of VOC emissions across maize genotypes in conferring indirect pest defense and provide evidence that these VOCs’ defense traits may be lacking in some genotypes. As such, these defense traits should be exploited in producing pest-resistant crop varieties for agricultural sustainability because they have better attraction of parasitoid wasps when crops are damaged by FAW (Stenberg et al. 2015).

**Statements and Declarations**.

## Data Availability

No datasets were generated or analysed during the current study.
